# Antibacterial efficacy of AH Plus and AH26 sealers mixed with amoxicillin, triple antibiotic paste and nanosilver

**DOI:** 10.15171/joddd.2016.035

**Published:** 2016-12-21

**Authors:** Ali Kangarlou, Rojin Neshandar, Negin Matini, Omid Dianat

**Affiliations:** ^1^Department of Endodontics, Dental School, Shahid Beheshti University of Medical Sciences, Tehran, Iran; ^2^Private Dental Practice, Tehran, Iran

**Keywords:** Antibacterial, root canal sealer, *Enterococcus faecalis*, antibiotic, agar diffusion test

## Abstract

***Background.*** Elimination of bacteria from the root canal system is one of the aims of endodontic treatment; hence the incorporation of antibiotics into sealers can increase their antimicrobial efficacy. The aim of the present study was to determine the in vitro antimicrobial effects of AH26 and AH Plus sealers mixed with amoxicillin, triple antibiotic paste and nanosilver on *Enterococcus faecalis*.

***Methods.*** In this experiment, amoxicillin, triple antibiotic paste and nanosilver powder were added at 10% of the total sealer weight to AH26 and AH Plus sealers and then cultured freshly or after 1, 3, and 7 days with suspension of E. faecalis for 24 hours. The zones of growth inhibition for *E. faecalis* were evaluated in each group.

***Results.*** Incorporation of nanosilver did not increase antibacterial effects of the sealers. Sealers combined with amoxicillin exhibited the highest antibacterial efficacy in fresh condition. In the set specimens, the results demonstrated that the mixture of sealers and triple antibiotic pastes exhibited the greatest antibacterial efficacy.

***Conclusion.*** Amoxicillin and triple antibiotic paste significantly improved the antibacterial properties of AH Plus and AH26 sealers. Such properties decreased with time, but the use of sealer-amoxicillin/triple paste combination was still superior to using sealers alone or in combination with nanosilver.

## Introduction

The primary etiologic factors for pulpal necrosis and periapical lesions are bacteria and their by-products.^1^ Persistence of intraradicular infections and secondary infections are the main reasons for endodontic failures.^2-5^ During endodontic treatments, efforts are made to minimize the number of microorganisms through mechanical and chemical procedures.^6,7^ However, there is always a possibility that some still remain in the canals.

*Enterococcus faecalis*, a gram-positive facultative anaerobic microorganism and part of the normal flora of the mouth, might be found in small amounts in unfilled root canals. Invasion of dental tubules, resistance against various ecological conditions and compatibility with the inappropriate conditions inside the root canals are among the factors that introduce this microorganism as a resistant pathogen in endodontic treatments.^8^

Endodontic sealers are applied to prevent periradicular exudates from leaking into the unfilled areas of the root canal and to prevent the remaining microorganisms from reaching the periapical tissues by eliminating the spaces between the obturation material and root canal walls.^9^ A good sealer should have properties such as favourable tissue tolerance, no shrinkage on setting and insolubility in oral and tissue fluids. Also, inhibition of microbial growth can be an optimal property for a sealer.^10^ AH26 is the most commonly used epoxy resin-based sealer. Some level of antibacterial activity was found as one of the properties of AH26.^11-13^Both AH26 and AH Plus have gained popularity as root canal sealers.

Triple antibiotic paste (TAP), containing metronidazole, ciprofloxacin and minocycline, has been reported to be an effective combination in eradicating root canal bacteria, and it has been found to be effective in vitro and in vivo.^14,15^

Some studies have evaluated the antimicrobial effects of various antibiotics such as amoxicillin, vancomycin, erythromycin, benzylpenicillin and doxycycline against *Enterococcus faecalis*.^16-18^ When used locally, the side effects of systemic antibiotic administration are prevented and a higher concentration of the medicament is available.^19^ In 2006, Hoelscher et al showed that sealer-antibiotic combinations containing amoxicillin, penicillin, clindamycin and doxycycline can significantly increase the zones of growth inhibition when compared to sealers alone.^20^ Also, Baer and Maki (2010) reported that sealers mixed with amoxicillin had a significantly greater inhibitory effect on the growth of *Enterococcus faecalis* as compared to sealers without amoxicillin.^21^ It seems that the adding antibiotics to sealers can increase their antimicrobial efficacy and inhibit the growth of the remaining microorganisms in the root canal. However, antibiotics have not improved the sealing ability and the physical properties of endodontic sealers^22^ and might result in antibiotic resistance.^23,24^

Nanosilver is one of the recent nano-technological products with some antibacterial characteristics. The silver attaches to bacterial cell walls, adversely affects cellular metabolism and inhibits cell growth.^25^ The incorporation of nanosilver into root canal medicaments resulted in less bacterial colonization in the root canal system.^26^

To date, no study has evaluated the incorporation of amoxicillin, triple antibiotic paste and nanosilver as root canal medicaments into AH Plus and AH26 sealers.

The aim of the present study was to determine the in vitro antimicrobial effects of AH26 and AH Plus sealers mixed with amoxicillin, triple antibiotic paste or nanosilver on *Enterococcus faecalis*.

## Methods

This in vitro study evaluated the culture results and measured the zones of growth inhibition for *Enterococcus faecalis* in freshly mixed and set combinations of AH26 or AH Plus sealers with amoxicillin (AMX), triple antibiotic paste (TAP) and nanosilver (NS) powder after 1, 3 and 7days.

AH26 (De Trey, Dentsply, Konstanz, Germany) and AH Plus (De Trey, Dentsply, Konstanz, Germany) sealers were prepared and mixed according to the manufacturer’s instructions. In experimental groups, amoxicillin, nanosilver and TAP were added at 10% of the sealers’ total weight. For TAP, a mixture of equal amounts of metronidazole (Metronidazole HCL; Alborz Darou, Iran), ciprofloxacin (Ciprofloxacin HCL; Arya Pharmaceuticals, Tehran, Iran), and minocycline (Minocin, Watson Pharmaceuticals Inc., California, USA) powder was added to each sealer at 10% of the sealer’s total weight. The amount of antibiotic was determined based on the results of previous studies.^20,21^

A standard strain of *Enterococcus faecalis* (ATCC 29212) was obtained from Pasteur Institute (Tehran, Iran). In order to ensure purity, a culture of bacteria was grown on a specific culture medium and evaluated under a microscope. A specific amount of pure colonies grown on the blood agar culture medium was transferred and cultured in 10 mL of TSB (Trypticase Soy Broth). After incubation for 24 hours at 37°C, an 0.5 McFarland standard suspension was prepared (one mL of suspension contained 1.5×10^8^ bacteria). For each inoculation, bacterial suspension was prepared with the same method and similar concentration to match the conditions.

Bile esculin culture medium was then prepared and *Enterococcus faecalis* was grown on it after procurement with the standard method. Eight paper discs measuring 6mm in diameter were saturated with AH Plus, AH Plus + AMX, AH Plus + TAP, AH Plus + NS, AH26, AH26 + AMX, AH26 + TAP and AH26 + NS, and then freshly placed on the culture media. One plain disc without the addition of any sealer and antibacterial agent was selected as the control. The plates were stored in an incubator for 24 hours at 37°C and the zone of inhibition around each paper disc was determined.

In the next phase, eight experimental groups were prepared and set for 1 day, 3 days, and 7 days in an incubator at 37°C. After the setting, the samples were transferred to a culture medium and stored for another 24 hours in an incubator at 37°C. The results were evaluated after completion of this time period. Data were analysed with SPSS 16. The diameters of the inhibition zones were measured and recorded in each experimental group, with the diameter of 6 mm as the cut-off value. The comparison of the inhibition zones in freshly mixed and set samples after 1, 3 and 7 days of incubation was carried out by using Kruskal-Wallis test; two-by-two comparisons of the samples were carried out with Dunn’s test.

## Results

The mean inhibition zone diameter in freshly mixed and set combinations has been summarized in Table 1.

**Table 1:** The mean inhibition zone diameter in freshly mixed and set combinations

**Groups**	**Time**	**Min**	**Max**	**Mean ± SD**
**AH Plus + Nanosilver**	Fresh	10.0	10.0	10.0±0
	1day	0	0	0
	3days	0	0	0
	7days	0	0	0
**AH Plus**	Fresh	10.0	10.0	10.0±0
	1day	0	0	0
	3days	0	0	0
	7days	0	0	0
**AH Plus + Amoxicillin**	Fresh	31.0	33.0	32.0±1.0
	1day	11.0	12.0	11.5±0.707
	3days	12.0	13.0	12.5±0.707
	7days	0	0	0
**AH Plus + Triple paste**	Fresh	18.0	21.0	20.0±1.73
	1day	15.0	19.0	17.0±2.83
	3days	19.0	20.0	19.5±0.707
	7days	19.0	20.0	19.5±0.707
**AH26 + Nanosilver**	Fresh	21.0	23.0	22.0±1.0
	1day	16.0	18.0	17.0±1.41
	3days	12.0	12.0	12.0
	7days	0	0	0
**AH26**	Fresh	23.0	27.0	25.0±2.0
	1day	17.0	17.0	17.0
	3days	13.0	14.0	13.5±0.707
	7days	0	0	0
**AH26 + Amoxicillin**	Fresh	36.0	39.0	37.33±1.53
	1day	21.0	24.0	22.5±2.12
	3days	21.0	21.0	21.0
	7days	22.0	24.0	23.0±1.41
**AH26 + Triple paste**	Fresh	29.0	32.0	30.33±1.53
	1day	20.0	23.0	21.5±2.12
	3days	24.0	26.0	25.0±1.41
	7days	22.0	23.0	22.5±0.707

The comparison of the diameters of growth inhibition zones in various freshly mixed and set combinations for 1, 3 and 7 days revealed significant differences among them (freshly mixed, P < 0.002; 1 day, P < 0.048; 3 days, P < 0.039; and 7 days, P < 0.04).

In this study, freshly mixed ‘AH 26 + AMX’ exhibited the greatest zone of growth inhibition among all the other studied groups (P < 0.05). ‘AH Plus + NS’ group and ‘AH Plus’ group exhibited the least zone of inhibition against the studied bacteria. Three groups that exhibited zone of growth inhibition even on day 7 were as follows: ‘AH Plus + TAP’, ‘AH 26 + TAP’, and ‘AH 26 + AMX’.

The paired comparison of the samples in terms of their antimicrobial efficacy using Dunn’s test revealed the following:

Freshly mixed samples:

AH Plus = AH Plus + NS < AH Plus + TAP < AH26 + NS < AH26 < AH26 + TAP < AH Plus + AMX < AH26 + AMX

Set specimens after 1 day:

AH Plus = AH Plus + NS < AH Plus + AMX < AH Plus + TAP = AH26 + NS = AH26 < AH26 + TAP = AH26 + AMX

Set specimens after 3 days:

AH Plus + NS = AH Plus <AH26 + NS = AH Plus + AMX < AH26 <AH Plus + TAP <AH 26 + TAP = AH26 + AMX

Set specimens after 7 days:

AH Plus + NS = AH Plus = AH26 + NS = AH26 = AH Plus + AMX < AH Plus + TAP < AH26+ TAP = AH26 + AMX

According to this experiment, AH26 + amoxicillin exhibited the greatest antibacterial effect against *Enterococcus faecalis,* while AH Plus and AH Plus + nanosilver exhibited the least effect (P ≤ 0.05).

## Discussion

Many studies have demonstrated that anaerobic bacteria play an important role in persistent root canal infections. These bacteria have a survival potential in necrotic environment with a lack of blood and oxygen. Facultative anaerobes may interact with anaerobes and influence their nutrition. The condition in endodontic treatment makes it mandatory to use root canal sealers with antibacterial characteristics.^5,27,28^

*Enterococcus faecalis* was selected for this study because it is difficult to eliminate this bacterium from root canal system during endodontic treatments.^21,29,30^* Enterococcus faecalis* can result in periapical inflammation^3,4^and it has the ability to survive even after the application of conventional antimicrobial agents like alkaline pH of calcium hydroxide.^31-33^

The agar diffusion test is one of the most commonly used methods in experiments regarding the antibacterial effects of root canal sealers in vitro.^12,13,34^ However, it has some limitations such as plate conditions in laboratory, agar viscosity, size and number of specimens in each plate, time and temperature of the existing plates and lack of standardization of inoculum density.^35^ In order to gain more conclusive results and exclude the variables, we have standardized the factors mentioned above.

This study demonstrated that incorporation of nanosilver into AH Plus did not significantly increase the mean zone of inhibition when compared to the sealer alone. However, the incorporation of amoxicillin and triple paste increased the antimicrobial efficacy of the sealer in both freshly mixed and set specimens.

For AH26 sealer, the incorporation of triple paste and amoxicillin similarly increased its antimicrobial efficacy. Considering the findings mentioned above, AH26 has a greater antimicrobial effect than AH Plus (both separately and in combination with antibiotics). This difference may be due to the release of formaldehyde by AH26 during the time intervals mentioned.^29^

Baer and Maki (2010) reported that sealer‒amoxicillin combinations kept their antimicrobial activity even after 7 days and inhibited the growth of *Enterococcus faecalis*.^21^ Sealers without amoxicillin could not prevent the growth of this microorganism. These findings were consistent with those of the current study. However, Baer and Maki used EWT (zinc oxide eugenol-based sealer), AH Plus (epoxy resin-based sealer) and RealSeal SE (polymethacrylate resin-based) sealers. They added antibiotics at 10% of the sealers’ total weight, similar to the current study. The selected amounts of antibiotics in both this study and that of Maki were based on the results of Hoelscher et al, who demonstrated that amoxicillin at 10% weight was the most effective in terms of antimicrobial properties.^20^ Also, in comparison to other antibiotics, amoxicillin had greater antimicrobial effects. Other reasons for selecting amoxicillin were its extended spectrum of bactericidal activity against many gram-positive and gram-negative microorganisms, its inexpensiveness, availability and fewer side effects as compared with other antibiotics.

In this study, triple paste was selected as an antibiotic because of its higher antimicrobial activity.^25^ Hoshino et al^36^ reported that TAP was effective at a concentration of 25 mg/mL of each antibiotic. Sabrah et al showed that TAP was effective at a concentration of 100 mg/mL of each antibiotic.^15^ These studies did not use antibiotics as an additive for sealers. We have added antibiotics to sealers, which was similarly carried out by Baer et al and Hoelscher et al. The different methodologies used can explain the differences between the results of previous studies.^20,21^

Nanosilver is safe and reportedly retains its antimicrobial efficacy over time. It is easily soluble in water and organic solvents, and has a high contact area due to its small particle sizes, resulting in good tissue compatibility. In addition, when mixed with other materials, it does not affect their properties.^37^

AH26 and AH Plus are both epoxy resin-based sealers, but AH26 releases formaldehyde over time, resulting in greater antimicrobial effects.^22^

Based on the results of the present study, AH Plus alone or in combination with nanosilver in set specimens after 1, 3 and 7 days did not show antimicrobial effect against *Enterococcus faecalis*. However, the freshly mixed AH Plus without antibiotics showed antimicrobial activity against *Enterococcus faecalis*. The above results are similar to those of Zhang et al (2009), who used modified Direct Contact Test (DCT) culture method and reported that after 1 day, AH Plus and Epiphany SE lost most of their antimicrobial activities.^38^ Researchers also showed that freshly mixed iRoot SP, AH Plus and EndoRez sealers are effective against *Enterococcus faecalis* strains.^38^ In 2006, Pizzo et al reported that freshly mixed AH Plus, Indometacin and pulp canal sealer completely inhibited the growth of *Enterococcus faecalis*.^39^ Some other researchers using direct method reported that eugenol-based sealers without the antibiotics had antimicrobial effects.^30,39-41^ The sensitivity of *Enterococcus faecalis* against antibiotics has been examined in several studies and it has been demonstrated that this microorganism is most susceptible to amoxicillin.^17,18^ However, some authors have reported this microorganism to be resistant to antibiotics such as ampicillin, clindamycin, and metronidazole.^16^ These differences in resistance pattern may be due to the strain of *Enterococcus faecalis*, various treatment protocols and the entity of endodontic infection (primary infection versus secondary infection).^16-18^

The incorporation of antibiotics to sealers may be effective in endodontic treatments.^42^Undoubtedly the application of sealers is critical for achieving a good seal and can decrease the rate of leakage in sealed canals5‒20 times.^43^ Amoxicillin is a broad-spectrum β-lactam bactericidal antibiotic. After mixing with sealers, its antimicrobial property persists after setting and it is especially important in the outcome of endodontic treatment and in preventing infection. Amoxicillin and triple antibiotic pastes have a significant effect on inhibiting *Enterococcus faecalis* based on the results of the present study. A study by Adl et al showed that triple antibiotic paste was more effective in eliminating *Enterococcus faecalis* from the root canal as compared to calcium hydroxide.^44^ In this study, the use of a small amount of antibiotic was sufficient as an antibacterial medicament in root canal sealers. However, it should be noted that the increased amount of local administration of antibiotics may be toxic for host cells and a limited concentration is recommended. Further investigations are required to evaluate the exact amount of antibiotic needed in root canal sealers as well as the setting time, shrinkage and solubility and discoloration potential of the sealers.

Despite the importance of in vitro studies, care should be taken when attributing the results to the clinical settings due to the presence of confounding factors related to both the patient and dentist that are hard to control. On the other hand, due to the complexity of the root canal system, complete elimination of bacteria is not possible in many circumstances.^45^ Some studies demonstrated that the presence of the smear layer prevents the sealers from sufficient penetration into the dentinal tubes.^46,47^ Therefore, the root canal system can affect the antibacterial activity of different medicaments.^48^

## Conclusion

In conclusion, the present study demonstrated that amoxicillin and triple antibiotic paste can significantly improve the antibacterial properties of AH Plus and AH26 sealers. Such properties usually decrease with time, but the use of the sealer‒amoxicillin/triple paste combination is still superior to using sealers alone or in combination with nanosilver.

## Acknowledgments

The authors thank Microbiology Department of Shahid Beheshti Medical School for its assistance in this research.

## Authors’ contributions

AK, and OD contributed to the concept and design of the work. RN and NM conducted lab tests and acquisition of data. OD and NM drafted manuscript and revised it critically for intellectual content. All the authors read and approved the final manuscript.

## Funding

This project was supported and funded by the authors.

## Competing interests

The authors declare no competing interests with regards to the authorship and/or publication of this article.

## Ethics approval

Not applicable.
